# Knowledge, attitudes and practices regarding urinary schistosomiasis among adults in the Ekombe Bonji Health Area, Cameroon

**DOI:** 10.11604/pamj.2018.29.161.14980

**Published:** 2018-03-19

**Authors:** Laura Ngolere Folefac, Peter Nde-Fon, Vincent Siysi Verla, Michael Nkemanjong Tangye, Anna Longdoh Njunda, Henry Namme Luma

**Affiliations:** 1 Department of Medicine, Faculty of Health Sciences, University of Buea, South West Region, Cameroon; 2 Department of Public Health and Hygiene, Faculty of Health Sciences, University of Buea, South West Region, Cameroon; 3 Department of Zoology and Animal Physiology, Faculty of Science, University of Buea, South West Region, Cameroon; 4 Department of Medical Laboratory Sciences, Faculty of Health Sciences, University of Buea, South West Region, Cameroon; 5 Department of Microbiology, Infectious Diseases, Faculty of Medicine and Biomedical Sciences, University of Yaoundé, Central Region, Cameroon

**Keywords:** Urinary schistosomiasis, knowledge, attitudes, practices, Ekombe Bonji

## Abstract

**Introduction:**

Urinary schistosomiasis (US) is endemic in Cameroon. Knowledge, attitudes and practices (KAP) are important aspects for control of the disease. However, data on these remain scanty. We aimed at evaluating knowledge, attitudes and practices regarding urinary schistosomiasis among adults in households in the Ekombe Bonji health area.

**Methods:**

A community-based, cross-sectional study was carried out at Ekombe Bonji health area from February to March, 2017, involving all 12 communities. A pre-tested questionnaire was used to assess knowledge, attitudes and practices regarding urinary schistosomiasis among 198 adults and to record their socio-demographic, environmental and clinical variables. Data were stored in Excel version 2013 and analysed using Stata version 14.2.

**Results:**

Of the 198 adults interviewed, only 35.4% had prior knowledge about urinary schistosomiasis. Among these, 94.3%, 74.3%, 57.7% knew the signs and symptoms, modes of transmission and preventive measures respectively. Only 14.3% knew the cause and treatment. 81.2% considered urinary schistosomiasis a serious disease and 77.1% believed it could be prevented, albeit, their practices to prevent infection were inadequate.

**Conclusion:**

Knowledge, attitudes and practices regarding urinary schistosomiasis among adults are inadequate, since most of them are not aware of the disease. Therefore, there is need for community-based interventions especially health education to effectively reduce the disease burden.

## Introduction

Schistosomiasis remains a major public health problem, affecting almost 240 million people worldwide especially those in poor communities without access to safe drinking water and adequate sanitation [1], 85% of whom live in sub-Saharan Africa [2]. It is estimated that 20,000-200,000 people die of schistosomiasis each year [3]. Schistosomiasis (also known as bilharzia) is a parasitic disease caused by blood flukes of the genus Schistosoma [3]. Six Schistosoma (S.) species cause infection in humans: S. haematobium (causing urinary schistosomiasis), S. mansoni, S. japonicum, S. intercalatum, S. mekongi and S. guineensis (causing intestinal schistosomiasis) [4]. In Cameroon, schistosomiasis is unevenly distributed and involves 3 species: S. haematobium, S. mansoni and S. intercalatum, with S. haematobium being the most predominant [5]. Urinary schistosomiasis (US) is a chronic disease, affecting over 112 million people in sub-Saharan Africa [6]. Studies have shown that the disease is endemic in Cameroon [7, 8], with the South West Region being one of the most affected regions [9]. People get infected when the infective cercarial larvae of S. haematobium shed by infected Bulinus snail hosts penetrate the skin during contact with water [10, 11]. The main strategy for the control of US in Cameroon is the National Deworming program using praziquantel administered to school children as advocated by World Health Organisation (WHO) [12]. This is limited in the fact that older population segments are insufficiently addressed, and hence new knowledge on prevention and control of schistosomiasis infections is minimal [6]. In designing and implementing effective community based deworming programs, knowledge, attitudes and practices (KAP) of targeted people were found to be instrumental [13]. Furthermore, community awareness or knowledge is considered one of the fundamental tools for the success and sustainability of any disease control program [14]. Also, many researchers in schistosomiasis appreciate the need to incorporate social science research in understanding the disease and designing more effective control interventions [8-9, 15-24], yet only a few have actually incorporated social aspects into their studies. In Cameroon, residents of rural areas related haematuria to excessive exposure to sunlight and sexual intercourse thereby dismissing medical treatment in local hospitals [25, 26], while others related it to puberty and eating of unclean fruits [25]. Against this background, this study aimed at assessing the knowledge, attitudes and practices regarding US among adults in households in the Ekombe Bonji health area. Findings from this study will provide baseline information on knowledge, attitudes and practices regarding US in the Ekombe Bonji health area. These will be useful in future assessments, as the information can help subsequent interventions in the control of the disease and most importantly, the control strategies will be based on the community needs.

## Methods

**Study design:** This was a cross-sectional, community-based study, involving all 12 communities. The study was carried out over a period of 7 weeks. After obtaining administrative and ethical clearances, visits were made to the village authorities to explain the procedures and benefits of the study, as well as plan dates for visits to households. 255 households were randomly selected for this study. During the household survey, the study was explained to the participants. Unique study numbers were assigned to each household and to each household head. A structured questionnaire was used to collect data on socio-demographic, environmental and clinical variables of the adults.

**Study setting:** The study was carried out from 6^th^ February to 31^st^ March, 2017, in the Ekombe Bonji health area which comprises of 12 rural communities in the Kumba health district. It is located 10.4km from Kumba and shares boundaries in the South with Kotto Barombi Health Area (the location of one of the two transmission sites for schistosomiasis [**5**]). The estimated population is 9000, with about 706 houses. It has three government integrated health facilities: Ekombe Bonji Health Centre as the main one, Small Ekombe and Bai Manya Health Centres, as well as private health institutions. The health area has many water bodies (4 rivers and 15 streams) as well as portable water which is not easily accessible because of distance and irregular flow of water. Also, some communities completely lack the supply of portable water. Therefore, a great proportion of the inhabitants tend to use natural water bodies for their daily activities like bathing, washing of clothes and dishes. Most inhabitants are of the Oroko tribe. Other ethnic groups include: The Bangwa, Bamileke and Ibo. The main occupation of the people is farming and the crops grown are cocoa and local crops. Other occupations include: Fishing, tailoring, hairdressing, teaching and trading. One of the main food stuff traded is snails, which happen to be the intermediate host of the schistosomes. Most of the people practice Christianity, few practise Islamism and others are either traditionalists (referring to those who did not hold on to God, but rather believed in tradition and practised idol worshipping) or pagans (referring to those who believe in God but do not follow any particular religion, neither do they practise idol worshiping; they prefer to pray at home rather than join any congregation). This health area was selected because despite the fact that many studies had been conducted in neighbouring places like Kumba [27], no study has been carried out in the health area as a whole. Also, as earlier mentioned, it shares boundaries with Kotto Barombi, a prominent and well documented focus of infection [5].

**Study population:** Participants in this study were household heads or any adult representative available during the household survey and who was an inhabitant of the health area. Only one adult was interviewed per household.

**Questionnaire survey:** A semi-structured questionnaire was adapted from a study conducted in Yemen [28]; the questionnaire was slightly modified, piloted and used to obtain information on knowledge (about US; its signs and symptoms, cause, mode of transmission, prevention and treatment), attitudes and practices regarding US. The questionnaire was also used to collect socio-demographic data including age, gender, name of community, highest level of education attained, occupation, environmental factors concerning portable water, number of water bodies used in the locality and the history of US infection. Questions on knowledge were open-ended questions, without multiple-choice answers to avoid guessing, which might have given a false impression concerning the knowledge of the participant. However, the questions pertaining to the practices were provided with multiple-choice answers to assess whether or not they performed these activities. Similarly, questions on attitudes were closed-ended questions. The questionnaire was chosen from Yemen because it was the most complete when compared to many others. In addition, the study population was the same in both studies. The questionnaires were pretested and administered to the household heads through face-to-face interviews. The total number of questions were 30 (K-5, A-4, P-8, others-13). In the absence of household heads, an adult representative was interviewed. Questionnaires were administered in English or pidgin English, a local lingua franca, widely spoken in the West African coast.

**Statistical analysis:** Data were coded, classified, tabulated and then entered into Excel version 2013, from where it was imported unto Stata version 14.2 for analysis. Before analysis, data were printed out and cross-checked for missing information, inconsistency and for any outliers. Frequency and cross-tabulation tables were computed. Schistosomiasis knowledge questions were computed. 1 point was allocated for a correct response followed by a point of 0 for a “don't know" or a wrong answer, with a total score of 5. A score of < 2 was considered poor, 2-3 average and ≥4 good. Attitudes responses were computed. 1 point was allocated for a positive attitude (meaning those who had the right belief about the disease), followed by a point of 0 for a “don't know" response (meaning those who were indifferent about the question) or a negative attitude, with a total score of 4. A score of < 2 was considered poor, 2 average and ≥3 positive attitude. Responses to questions on practices were computed. 1 point was allocated for a favourable practice and 0 for an unfavourable (risky) practice, with a total score of 8. A score of < 4 was considered poor, 4-6 average and > 6 favourable. The Chi-square (χ^2^) test was used to test the association between KAP and the socio-demographic and clinical variables. A p-value of < 0.05 was considered to be statistically significant.

**Ethical approval:** Ethical approval was obtained from the Institutional Review Board of the Faculty of Health Sciences, University of Buea (2017/003/UB/SG/IRB/FHS). Administrative clearances were obtained from the Faculty of Health Sciences, University of Buea (2017/336/UB/VD/RC/FHS), the Regional Delegation of Public Health for the South West Region, Cameroon (R11/MINSANTE/SWR/RDPH/PS/40/679) and from the Kumba Health District (02/2017/MINSANTE/RDPHSW/KHD/DMO/01). In the health Area, authorisations were obtained from the heads of the various communities. During the household survey, consent-informed verbal and written, was sought from the adults using consent forms that carried information about the study. The study was well explained to all eligible adults in simple English language and pidgin English. Only those who consented were enrolled into the study. Participants were identified using unique study numbers instead of names and all information obtained from the participants was treated as confidential.

**Study limitations:** Some participants did not understand some key terms like urinary schistosomiasis, making the administration of questionnaires difficult. We had to meet with some traditional rulers to get the meaning in the “Oroko" vernacular. It was a challenge trying to get questions answered in terms of the time it took, patience and hoping that all translations reflected the true meaning. Also, our sample size for evaluating knowledge and attitudes (70) was small.

## Results

**Socio-demographic characteristics of the study population:** From the 12 communities in Ekombe Bonji health area, 255 households were visited but only a total of 198 household heads were interviewed face-to-face to fill in the questionnaire on their knowledge, attitudes and practices towards US. The remaining 57 household heads either did not give their consent (48) or opted out of the study (9) and so were not included in the study. Of the 198, 136 (68.7%) were females and 62 (31.3%) were males. The mean age of the participants was 36 ± 2.3 years with the ages ranging from 18-80 years. Participants were further divided into 2 age groups: < 40 (128) and ≥40 (70). Majority (141, 71.2%) of the householders had attained primary education (at least 6 years of formal education), 27(13.6%) of them had attained secondary education, and 15(7.6%) of them had attained post-secondary education while 15(7.6%) householders had no formal education. A very small proportion (4.6%) had a history of US infection.

**Knowledge and attitudes regarding urinary schistosomiasis:** It was found that only a minority: 35.4% (70/198) of respondents had heard about US. Of these, 55.7% got the information from home (family and neighbours) and 14.3% (10/70) of them knew the cause. 74.3% (52/70) mentioned at least one mode of transmission (bathing or contact with infected water), although no one mentioned the role of snail vectors. Furthermore, 94.3% (66/70) mentioned at least one sign or symptom related to the disease and 55.7% (39/70) mentioned at least one measure of prevention. Regarding attitudes, 80.0% (56/70) considered it a serious disease and 77.1% (54/70) believed that it could be prevented (Table 1).

**Table 1:** Knowledge and attitudes regarding urinary schistosomiasis (n=70, N=198)

Variable	Category	Number n (%)	% of the total study population (N)
Knowledge: Source of Information	School	12 (17.1)	6.1
	Health Center	16 (22.9)	8.1
	Mass Media	2 (2.9)	1.0
	Home	39 (55.7)	19.7
	Researchers	1 (1.4)	0.5
Cause of US	Worms	10 (14.3)	5.1
	Bacteria	7 (10.0)	3.5
	Witchcraft	5 (7.1)	2.5
	Don’t know	48 (68.6)	24.2
Mode of transmission	Bathing in infected water	33 (47.1)	16.7
	Contact with infected water	19 (27.1)	9.6
	Sexual intercourse	7 (10.0)	3.5
	Don’t know	11 (15.7)	5.6
Signs and Symptoms of US	Bloody and Painful urine	9 (12.9)	4.6
	Bloody urine	56 (80.0)	28.3
	Painful urination	1 (1.4)	0.5
	Don’t know	4 (5.7)	2.0
Prevention	Avoid contact with infected water	33 (47.1)	16.7
	Don’t urinate in infected water	4 (5.7)	2.0
	Health education	2 (2.86)	1.0
	Others	4 (5.7)	2.0
	Don’t know	27 (38.6)	13.6
Treatment	Consult	18 (25.7)	9.1
	Worm medicine	10 (14.3)	5.1
	Traditional medicine	1 (1.4)	0.5
	Don’t know	41 (58.6)	20.7
Attitudes: Is US a serious disease?	Yes	56 (80.0)	28.3
	No	4 (5.7)	2.0
	Don’t know	10 (14.3)	5.1
Can it be prevented?	Yes	54 (77.1)	27.3
	No	2 (2.9)	1.0
	Don’t know	14 (20.0)	7.1

**Practices towards urinary schistosomiasis:** Table 2 depicts the practices towards US. Majority (69.2%, 137/198) of the respondents urinate in open water and only 9.1% (18/198) use water-proof clothing when in contact with open water. In terms of treatment-seeking behaviour, majority (85.4%, 169/198) consult for urinary symptoms.

**Table 2:** Practices towards urinary schistosomiasis (N=198)

Variable	category	Number N	% of the total study population
Urinate in fresh water or bush	Yes	137	69.2
	No	61	30.8
Wash dishes/dresses in open water	Yes	138	69.7
	No	60	30.3
Swim/bathe in open water	Yes	131	66.2
	No	67	33.8
Fetch water from streams/rivers	Yes	136	68.7
	No	62	31.3
Use water-proof clothing when in contact with water	Yes	18	9.1
	No	180	90.9
Consult for urinary symptoms	Yes	169	85.4
	No	29	14.6
Take traditional treatment for urinary symptoms	Yes	22	11.1
	No	176	88.9
Ignore urinary symptoms	Yes	11	5.6
	No	187	94.4

**Figure 1:**
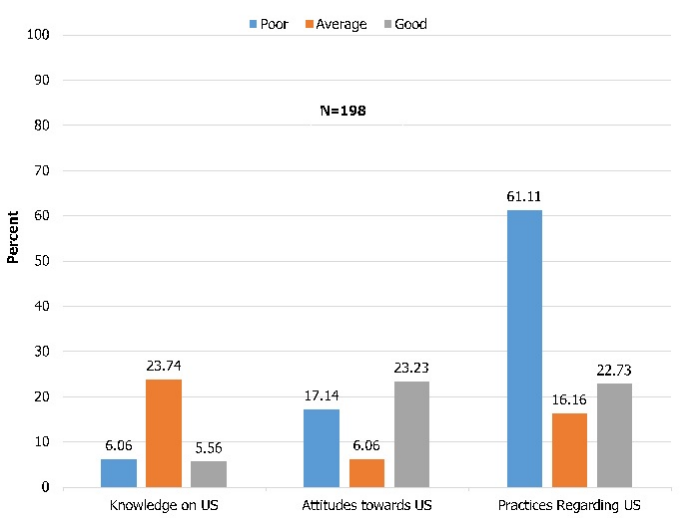
Participants' score on knowledge, attitude and practices

**Participants' score on knowledge, attitudes and practices:** As shown in Figure 1, only 5.56% of the study population had good knowledge on US, scoring 4-5/5 and 23.23% had good attitudes, scoring ≥3/4. Regarding practices, more than half (61.11%) of the study participants had poor practices, scoring < 4/8.

**Association between knowledge and some socio-demographic/clinical variables:** Of those who had heard about US (70), there was no significant association between knowledge on US and socio-demographic/clinical variables (Table 3).

**Table 3:** Association between knowledge and some socio-demographic/clinical variables (n=70)

Variable	Category	Poor knowledge n (%)	Average knowledge n (%)	Good Knowledge n (%)	p-value
Age	˂40	7 (17.07)	30 (73.17)	4 (9.76)	0.250
	≥40	5 (15.24)	17 (58.62)	7 (24.14)	
Gender	Male	3 (11.11)	18 (66.67)	6 (22.22)	0.347
	Female	9 (20.93)	29 (67.44)	5 (11.63)	
Religion	Christian	10 (14.93)	46 (48.66)	11 (16.42)	0.151
	Muslim	1 (100.00)	0 (0.00)	0 (0.00)	
	Traditionalist	1 (50.00)	1 (50.00)	0 (0.00)	
Level of Education	None	1 (50.00)	1 (50.00)	0 (0.00)	0.216
	Primary	10 (21.74)	31 (67.39)	5 (10.87)	
	Secondary	1 (9.09)	8 (72.73)	2 (18.18)	
	Post-secondary	0 (0.00)	7 (63.64)	4 (36.36)	
History of US	Yes	1 (11.11)	7 (77.78)	1 (11.11)	0.766
	No	11 (18.03)	40 (65.57)	(16.39)	

**Association of attitudes with some socio-demographic/clinical variables:** There was no significant association between the level of attitudes towards US and socio-demographic variables (Table 4).

**Table 4:** Association of attitudes with some socio-demographic/clinical variables (n=70)

Variable	Category	Poor Attitudes n (%)	Average Attitudes n (%)	Good Attitudes n (%)	p-value
Age	˂ 40	6 (14.63)	8 (19.51)	27 (65.85)	0.709
	≥ 40	6 (20.69)	4 (13.79)	19 (65.52)	
Gender	Male	7 (25.93)	5 (18.52)	15 (55.56)	0.257
	Female	5 (11.63)	7 (16.28)	31 (72.09)	
Religion	Christian	11 (16.42)	11 (16.42)	45 (67.16)	0.160
	Muslim	0 (0.00)	1 (100.00)	0 (0.00)	
	Traditionalist	1 (50.00)	0 (0.00)	1 (50.00)	
Level of education	None	0 (0.00)	1 (50.00)	1 (50.00)	0.310
	Primary	11 (23.91)	8 (17.39)	27 (58.70)	
	Secondary	1 (9.09)	2 (18.18)	8 (72.73)	
	Post-secondary	0 (0.00)	1 (9.09)	10 (90.91)	
History of US	Yes	3 (33.33)	2 (22.22)	4 (44.44)	0.294
	No	9 (14.75)	10 (16.39)	(68.85)	

**Association of practices with some socio-demographic/clinical variables:** Table 5 shows that of all the variables, only level of education was significantly associated with practices. Poor practices were significantly associated with a lower level of education and good practices with a higher level of education.

**Table 5:** Association of practices with some socio-demographic/clinical variables (N=198)

Variable	Category	Poor Practices	Average Practices	Good Practices	p-value
Age	˂ 40	82 (60.74)	23 (17.04)	30 (22.22)	0.553
	≥ 40	39 (61.90)	9 (14.29)	15 (23.81)	
Gender	Male	40 (64.52)	7 (11.29)	15 (24.19)	0.435
	Female	83 (61.03)	25 (18.38)	28 (20.59)	
Religion	Christian	118 (61.46)	31 (16.15)	43 (22.40)	0.218
	Muslim	0 (0.00)	1 (100.00)	0 (0.00)	
	Pagan	1 (100.00)	0 (0.00)	0 (0.00)	
	Traditionalist	4 (100.00)	0 (0.00)	0 (0.00)	
Level of education	None	11 (73.33)	2 (13.33)	2 (13.33)	0.000
	Primary	95 (67.38)	25 (17.73)	21 (14.89)	
	Secondary	14 (51.85)	3 (11.11)	10 (37.04)	
	Post-secondary	3 (20.00)	2 (13.33)	10 (66.67)	
History of US	Yes	4 (44.44)	2 (22.22)	3 (33.33)	0.529
	No	119 (62.96)	30 (15.87)	40 (21.16)	

## Discussion

The control of schistosomiasis in Cameroon can only be successful and sustainable if residents in and around the foci have the right knowledge, positive attitudes and correct preventive and control practices regarding the disease. Results from this study show that knowledge, attitudes and practices towards US among adults in Ekombe Bonji health area are generally inadequate because more than 60% of the study population do not know about US. This is similar to findings obtained by Sady et al [28]. On the contrary, Maseko et al [29] recorded moderate knowledge, good attitudes and fairly good practices regarding schistosomiasis in Swaziland. Only 35.35% of the respondents had heard about the disease. This may be because research on schistosomiasis had not been carried out before in the whole health area and therefore, health education, as a control strategy has not been instituted. Similar findings were obtained in Nigeria [30]. However, Sady et al [28] recorded higher level of awareness because their study area was undergoing active control of schistosomiasis at the time of study. We found that more than half of those who had prior knowledge on US got the information from home. Dawaki et al [31] obtained similar results and this could be a demonstration of the community's role in the disease control. However, Sady et al [28] recorded that majority of those who had prior knowledge got the information from the health centre. With regard to knowledge about signs and symptoms, 94.29% of those who had prior knowledge mentioned at least one sign or symptom, among which 80% mentioned haematuria. The local name for US in Oroko language is “miari ma makia" which means blood in urine, which may explain the good knowledge about haematuria. This finding is similar to that obtained in Swaziland [29] where 74% mentioned haematuria as a symptom. Other studies in Cameroon [27] and Yemen [28] have reported a lower level of knowledge about signs and symptoms of the disease as only 68% and 39.8% respectively, mentioned haematuria.

Only 14.29% mentioned worms as the cause of the disease. Similar results were obtained by Maseko et al [29] and Dawaki et al [31]. However, Kitalile et al [32] reported that 77.6% of respondents knew the cause of US. The difference may be due to the fact that in our study, questions on knowledge were open-ended, reducing the probability of guessing as opposed to the multiple-choice questions used by Kitalile et al [32]. Up to 74.29% of those who had prior knowledge mentioned at least one mode of transmission, however, no one mentioned the role of snail vectors. Also, 55.71% mentioned at least one preventive measure. On the contrary, Sady et al [28] reported that only 49.8% of respondents mentioned at least one mode of transmission and 11.3% mentioned the role of snail vectors. They also reported that 47.2% were able to give at least one measure of prevention. Midzi et al [33] also recorded that only 22.1% had knowledge on preventive measures. In addition, more than half of the respondents did not know how US is treated. Majority of those who had heard about US reported to have gotten the information from family members or neighbours. This may explain the poor knowledge on the cause, role of snail vectors in the mode of transmission and on the treatment. Our study showed that there was no significant association of knowledge on US with age, gender, religion, level of education and history of infection. This could be due to the fact that our sample size for assessment of Knowledge (n = 70) was small. Similarly, previous studies [32, 34] reported no significant association between educational level and knowledge on schistosomiasis. On the contrary, Dawaki et al [31] reported that age, gender, educational level and history of infection were significantly associated with knowledge. Sady et al [28] and Mwai et al [35] also reported that knowledge was significantly associated with sociodemographic factors: age and educational level.

Concerning attitudes towards US, 80.0% (56/70) believed it is a serious disease. Several people said that if a disease makes one to urinate blood, then it's serious. Similar results were obtained by Sady et al [28] and Dawaki et al [31]. Also, 77.14% of respondents believed that US can be prevented. However, in Yemen [28], less than half of the respondents believed it could be prevented. Although majority of respondents recognize the role of water bodies in the disease transmission, most risky practices were related to their water contact behaviour, as also observed by Maseko et al [29] and Abo-Madyan et al [36]. It could be due to the absence of pipe-borne water in some communities, leaving people with no choice but to use water bodies as their only source of water. This could be an obstacle to the eradication of the disease and may also indicate that awareness alone does not necessarily result in behavioural changes, which often require long periods of time to ensure compliance with healthier practices [37]. This clearly implies that provision of knowledge alone will not bring about significant changes in the foreseeable future. Therefore, there is urgent need for supply of portable water to communities in lack and to those with only few taps as this may go a long way to reduce their water contact behaviours. Also, provision of pit latrines may reduce the probability of urinating in fresh water. Furthermore, if these measures are not included in the control strategies, there is a risk of the people developing negative attitudes to control programmes as was the case in North-west Uganda [38]. Our results show a significant association between the educational level and practices regarding US, as also observed by Sady et al [28]. According to Adoka et al [39], education plays an important role in people's perceptions and practices of controlling schistosomiasis. This may explain the association between the level of education and practices of the people.

## Conclusion

This study reveals that knowledge, attitudes and practices towards US in the Ekombe Bonji health area are inadequate based on the fact that majority of the study population are not aware of the disease. This could be a challenging obstacle to the endeavour towards eliminating the disease. There is thus need for Intensive and continuous community-based health education to improve the level of awareness and knowledge on the disease, as it plays an important/vital role in ensuring an effective and sustainable control of the disease. In addition, there is urgent need for provision of pit latrines and supply of portable water to communities in lack and to those with only few taps. These may go a long way to reduce their water contact behaviours. We recommend that further studies on KAP regarding US be carried out in this area using focus group discussions, to elucidate the people's perceptions of the disease.

### What is known about this topic

- Knowledge on urinary schistosomiasis in studied areas in the southwest region is generally poor, as many do not know about the disease;
- Majority of the people studied still use surface water for their domestic activities.

### What this study adds

- This study provides baseline information on KAP regarding US in the Ekombe Bonji health area, and is a more detailed KAP survey on US, compared to other studies conducted in the Southwest Region of Cameroon;
- Though we assume that it is known, we find from our study that knowledge on US is still lacking and attitudes and practices are still inadequate, which is the reason for continued assessment, so that we can have more integrated and effective public health interventions;
- We also studied factors associated with knowledge, attitudes and practices.

## Competing interests

The authors declare no competing interests.
